# Are older people putting themselves at risk when using their walking frames?

**DOI:** 10.1186/s12877-020-1450-2

**Published:** 2020-03-04

**Authors:** Sibylle Brunhilde Thies, Alex Bates, Eleonora Costamagna, Laurence Kenney, Malcolm Granat, Jo Webb, Dave Howard, Rose Baker, Helen Dawes

**Affiliations:** 1 Centre for Health Sciences Research, School of Health & Society, University of Salford, Brian Blatchford Building Room PO28, Salford, Greater Manchester M6 6PU UK; 2 School of Computing, Science and Engineering, University of Salford, Salford, Greater Manchester UK; 3 School of Business, University of Salford, Salford, Greater Manchester UK; 4 Centre for Movement, Occupational and Rehabilitation Sciences, Faculty of Health and Life Sciences, Oxford Brookes University, Oxford, UK

**Keywords:** Older adults, Walking aids, Stability, Falls

## Abstract

**Background:**

Walking aids are issued to older adults to prevent falls, however, paradoxically their use has been identified as a risk factor for falling. To prevent falls, walking aids must be used in a stable manner, but it remains unknown to what extent associated clinical guidance is adhered to at home, and whether following guidance facilitates a stable walking pattern.

It was the aim of this study to investigate adherence to guidance on walking frame use, and to quantify user stability whilst using walking frames. Additionally, we explored the views of users and healthcare professionals on walking aid use, and regarding the instrumented walking frames (‘Smart Walkers’) utilized in this study.

**Methods:**

This observational study used Smart Walkers and pressure-sensing insoles to investigate usage patterns of 17 older people in their home environment; corresponding video captured contextual information. Additionally, stability when following, or not, clinical guidance was quantified for a subset of users during walking in an Activities of Daily Living Flat and in a gait laboratory. Two focus groups (users, healthcare professionals) shared their experiences with walking aids and provided feedback on the Smart Walkers.

**Results:**

Incorrect use was observed for 16% of single support periods and for 29% of dual support periods, and was associated with environmental constraints and a specific frame design feature. Incorrect use was associated with reduced stability. Participants and healthcare professionals perceived the Smart Walker technology positively.

**Conclusions:**

Clinical guidance cannot easily be adhered to and self-selected strategies reduce stability, hence are placing the user at risk. Current guidance needs to be improved to address environmental constraints whilst facilitating stable walking. The research is highly relevant considering the rising number of walking aid users, their increased falls-risk, and the costs of falls.

## Background

Fall-related injuries in older adults are a major and growing global health problem: 40% of older adults living at home experience a fall once a year [1], and in care homes, falls-rates are even higher [2]. The costs associated with falls is estimated at £2.3 billion/year [3], with falls resulting in significant impact on the life of the individual and their family [4]. ‘Walking’ is the core activity during which community-dwelling and care home-based residents can fall [5, 6]. To assist with upright balance and mobility, walking aids are commonly used. They are used indoors by 22% of UK older adults, and outdoors by 44% [7].

Paradoxically, self-reported use of a walking aid (“yes”/“no”) is a risk factor for falls [8]. However, such binary classification of an individual’s use or otherwise of a walking aid cannot capture the complex patterns of everyday use, and the exact underlying reasons for falls of walking aid users are to date unknown. The effectiveness of walking aids in preventing falls is at least partly determined by how they are used, yet we have limited information of how they are used in home environments or how frame use relates to fall occurrence. Further, the extent to which users receive guidance, and more importantly, whether or not they comply with guidance is poorly understood. One study found that over 80% of wheeled frame users reported to have not received any guidance on how to use their frame [9], and another reported that 66% of patients with hip pathology were not educated as to which hand to hold their stick in [10].

There are a number of leaflets, from both the NHS and manufacturers, which contain rather basic, brief but easy-to-follow instructions on how to use walking aids. For example, for a pick-up walker (a frame without wheels), correct use is described as: a) the walker being lifted forward only whilst the user is standing on both of their feet; and b) only when the walker is fully grounded again, should the user step towards it. The front-wheeled walker, by contrast, is not designed to be picked up, and clinical guidance, although varying somewhat between providers in the precise wording, states that the user should “glide the frame forward” on the ground (Oxford Health NHS Foundation Trust), or “push rather than lift the frame” (Trulife, a manufacturer). Considering these recommendations, and acknowledging that the rear legs do not have wheels and hence at times may need lifting, we infer correct use to be that the front wheels should remain grounded. However, leaflets generally fail to address how to use the frame when performing more complex tasks, for example crossing obstacles such as door thresholds and turning in confined spaces, despite users reporting a range of everyday tasks as problematic [11]. It therefore remains doubtful whether current guidance is adequately adhered to in the home environment, and whether following current guidance indeed facilitates safe everyday use of walking aids. This is supported by an observational study which reported inappropriate use in those recovering from hip fracture despite having received education on how to use their device [12]. The lack of understanding on how walking aids are used in everyday life is concerning, especially when considering the increasing number of users in our ageing population [7].

Only one study quantified walking aid use with an instrumented rollator (a four-wheeled walker with integrated load cells and video camera) both inside the gait laboratory and over a walking course within the building [13]. However, the findings were limited by the sample size (*n* = 3), are specific to use of a rollator frame, and did not include a thorough analysis of user-device stability. Specifically, the authors’ approach inferred stability of the (mechanically coupled) system of user and their rollator based on the forces placed onto the rollator alone, an approach which is inadequate. By contrast, we have shown that measurement of the forces through each of the walker’s feet and user’s anatomical feet, together with the relative location of each point of contact with the ground, is necessary to assess stability [14, 15].

Walking aid use remains poorly understood: how walking aids are used in home environments, and to what extent they are used in accordance with current guidance has not been quantified. In addition we do not know whether current guidance on use actually facilitates stable walking with a walking aid. Using our Smart Walker system [14], our aims therefore were to:

1. Investigate older adults’ use of walking frames in home settings (their home, residential home, care home, or sheltered housing) and compare to current guidance, and to explore associated contextual information, i.e. circumstances that may lead to deviation from guidance.
2. Investigate how older adults’ usage patterns affect their stability.

- Furthermore, as the introduction of new technologies into practice is always challenging, particularly in an area such as walking aids where measurement technology is rarely, if ever, used, we wanted to explore issues which may impact on the exploitation of our approach. Specifically, we wanted to understand end-users views on walking aid use in general, and with regard to the value of the Smart Walker system, their experiences of using the system, and how they would envisage engaging with the technology. Therefore, the project’s third aim was to:

3) Investigate the views of walking aid users and healthcare professionals on walking aids in general, and the Smart Walker System, specifically.

## Methods

### Design

An observational cross-sectional study, supported by focus group work.

### Participants

Inclusion criteria for participation were: 1) able to walk household distances with a walking frame, however, 2) not able to walk such distances repeatedly without a frame, 3) able to understand written and spoken English, able to follow a two-stage instruction. Exclusion criteria were: 1) currently in hospital, 2) visual disorders not correctable by glasses. A total of 17 older adults that were users of walking frames [age (mean ± SD): 70.3 ± 4.8, gender: 16 female & 1 male, body weight (mean ± SD): 78.1Kg ± 8.8Kg, Falls Efficacy Scale (mean ± SD): 42.5 ± 14.3] gave written informed consent and participated in the study. The Falls Efficacy Scale measures concerns about falling, with scores ranging from 16 (no concern) to 64 (severe concerns about falling). Participants lived in residential housing [3], sheltered housing [3], or care homes [11]. Ethical approval was sought and granted from the University of Salford’s Ethics Committee (HSCR16/35, HSCR13/48) and the London Dulwich Research Ethics Committee (16/LO/0986).

### Assessments

**Fig. 1:**
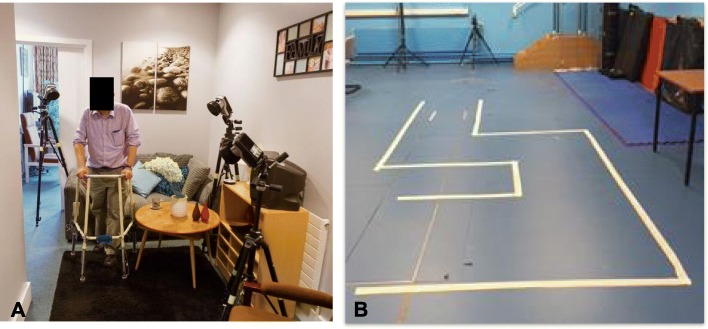
Data collection in (**a**) the ADL flat and (**b**) the gait laboratory. 3D camera data capture in the ADL flat provided a more realistic scenario, whilst the lab environment provided for clutter-free observation, enabling our stability metric to be calculated over a larger number of steps

Assessments took place in three environments: 1) ‘home’, which took place in their residential environment (e.g. home, care home or sheltered housing), 2) ‘ADL flat’ (Fig. 1a), which took place in the university’s Activities of Daily Living flat, which comprises a kitchen, bathroom, bedroom, and living room, and 3) ‘lab’, i.e. the university’s gait laboratory (Fig. 1b), where the walking pathways replicated from the ADL flat were marked on the floor, hence avoiding the distractions of furniture, carpet edges, door frames, door thresholds, and walls. Participants were given a choice to participate in any of these; this facilitated their recruitment as users of walking aids proved to be reluctant to commit to three assessments from the start, two of which required travel to the university. For each assessment participants were asked to walk common household distances (e.g. to walk from the lounge to the kitchen as if to make a cup of tea) with an instrumented pick-up walker and/or a front-wheeled walker, in accordance with what they generally use at home and set to their own walking frame’s height. Table 1 provides an overview of the participants’ assessments carried out in the three environments.

**Table 1:** Overview of assessments in the home, ADL flat, and gait laboratory. PW: pickup walker; FWW: front-wheeled walker

Participant	Home assessment		ADL flat assessment		Lab assessment	
	PW	FWW	PW	FWW	PW	FWW
1	X	X	X	X	X	X
2		X				
3		X				
4	X	X	X	X	X	X
5		X				
6		X		X		X
7		X		X		X
8		X		X		X
9		X		X		X
10		X				
11		X				
12		X				
13		X				
14		X				
15		X				
16		X		X		X
17			X		X	
Total	**2**	**16**	**3**	**7**	**3**	**7**

### Instrumentation

Two Smart Walker systems were used:

a) The “basic” Smart Walker System: this system consisted of four load cells, which recorded forces through each of the walker’s feet, and force-sensing insoles in the user’s shoes, which were synchronized with the load cells. The system transmits data, in real time, to a laptop. This set up allows for measurement of the periods during which the person’s feet or the frame’s feet/wheels are airborne. The data can also be used to calculate the distribution of body-weight support between the frame and each of the user’s feet, which is clinically important for an understanding of the support the person receives from the device, and to characterize weight-bearing on either leg. This system was used in users’ home environment where the use of 3D optoelectronic cameras was not feasible. However, video was recorded to provide information on the behaviours and/or environmental challenges faced by the user during any periods in which they may be using their frame incorrectly.

b) The “extended” Smart Walker System which, in addition to load cells and insoles as for the above, included synchronized 3D optoelectronic cameras. Those provide data on the relative position of the feet of user and frame, thereby also allowing for calculation of:

- The combined Base of Support (BoS) of the user-frame system, defined as the convex polygon formed by the boundaries of the anatomical and walking frame feet in contact with the ground and the interconnecting lines between them.
- The combined Centre of Pressure (CoP), the point through which the resultant ground reaction force for all feet of both the walking frame and user acts if the resultant moment acts only around an axis perpendicular to the ground plane.
- The combined stability margin ‘SM’ of the user-frame system, defined as the distance between the system’s CoP and the nearest edge of the BoS. From that, we compute the minimum value of the stability margin ‘SM_min_’ for each single or dual support period. SM_min_ occurs at the instant when the system is closest to “tipping over”. The position of each point of contact with the ground, derived from the camera data, is integral to quantification of stability, as it allows for user and frame to be treated as a single multi-legged moving system [15]

**Fig. 2:**
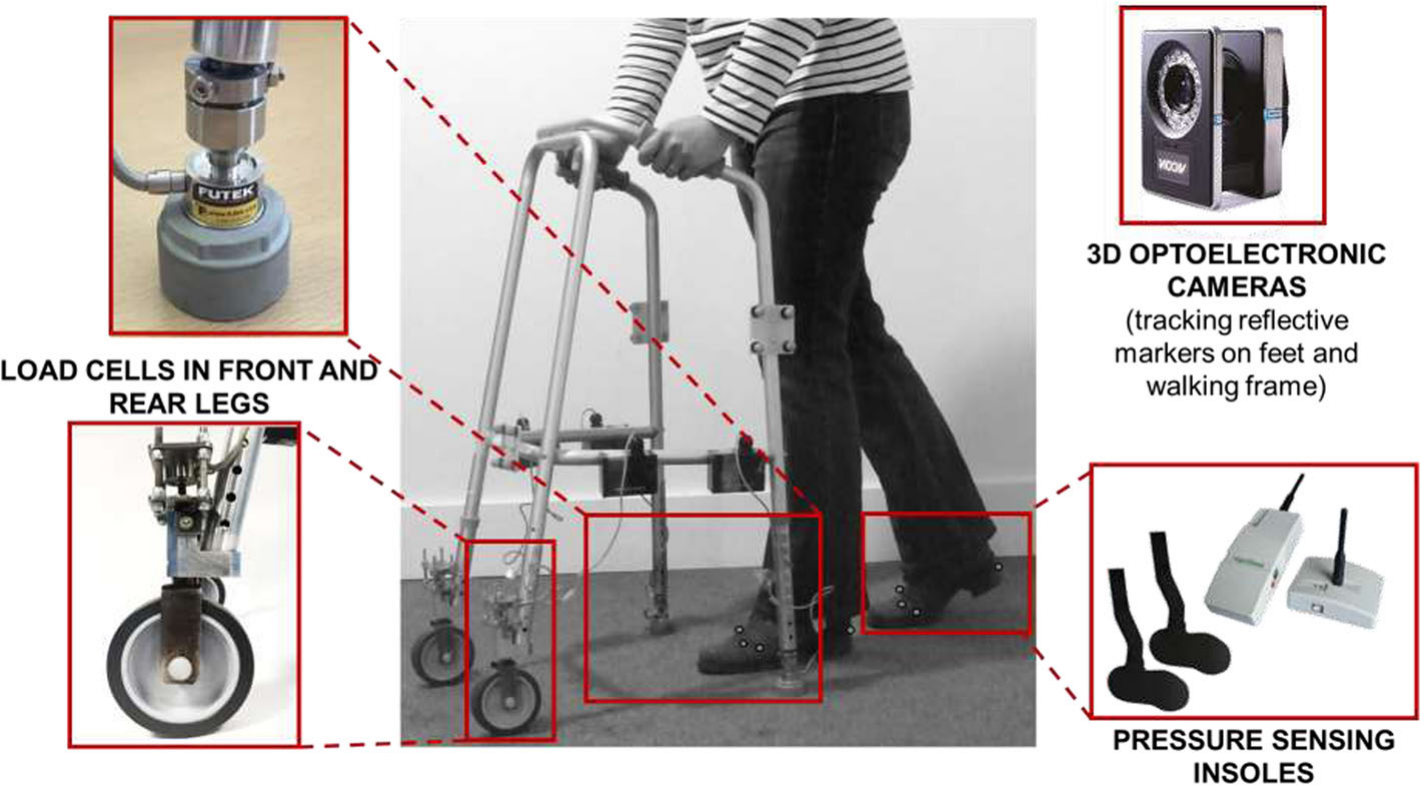
Illustration of all instrumentation for the example of a front-wheeled walker. The basic Smart Walker System only included load cells in the walking frame’s feet and force sensing insoles in the user’s shoes and was used in users’ homes. The extended Smart Walker System additionally included 3D optoelectronic cameras and was used in the university’s ADL flat and gait laboratory. The optoelectronic cameras recorded position data of reflective markers placed on the user’s feet and the frame. Note that whilst a front-wheeled walker is shown in this Figure, a basic and an extended pick-up walker system were also used in this study

The basic Smart Walker System was used for home assessments to identify if usage patterns in the real world are in accordance to what clinical guidance suggests to be safe, and also to characterize device loading in relation to the observed usage patterns. The extended Smart Walker System, which included 3D optoelectronic cameras, was used in the university’s ADL flat and gait laboratory to accurately quantify walking stability for the combined user-frame system in relation to the observed usage patterns. The assessment in the home environment was necessary to investigate older adults’ use of walking frames in home settings, but did not provide stability data; the assessments in the lab and ADL flat provided data on the users’ stability when walking in a wide space, free of clutter (gait lab), and in a more representative environment (ADL flat). Figure 2 shows the relevant instrumentation for the example of a “smart” front-wheeled walker system, however, a basic and an extended Smart Walker System was also developed for a pick-up walker. Camera data and load cells data were sampled at 100 Hz, pressure-insole data at 50 Hz. Data were aligned post data collection through resampling in Matlab®.

### Data analysis

For both frames, a given frame foot/wheel was considered ‘airborne’ if ground contact was lost for 10 sampling frames or longer. Moreover, considering that flooring is not perfectly flat, we considered the pick-up walker to be grounded when at least 3 of its 4 ft were in contact with the ground. Custom-written algorithms (MATLAB®) were used to compute:

1. The times when the walking frame feet were airborne (from load cell data).
2. The times when the user was in single and dual support (from pressure insole data).
3. Adherence to guidance, i.e. amalgamating information from 1 & 2 to calculate the number of single support and dual support periods where correct/incorrect use was the case and calculate % incorrect use. Based on clinical and manufacturer leaflets, correct use of a pick-up walker entails that the walker is lifted forward only whilst the user is standing on both of their feet, and incorrect is to step before the walker is fully grounded again. Correct use of the front-wheeled walker is to “glide the frame forward” on its front wheels (the rear feet may at times may need lifting), and incorrect would be to lift the front-wheels. We note that the guidance for safe use of pick-up walkers focuses entirely on dual support (“do not step whilst the walker is airborne”), hence % incorrect use of the pickup walker was only determined for single support periods (i.e. stepping whilst the walker is airborne). For the front-wheeled walker, incorrect use (lifting the front wheels) was determined for both, single and dual support periods.
4. The % body weight placed on the walking frame (i.e. ‘device loading’) for correct/incorrect use during single and dual support (from load cell data). And, for the extended Smart Walker system using also 3D optoelectronic cameras:
5. The minimum stability margin SM_min_ for correct/incorrect use during single and dual support (from insole, load cell, and camera position data).

**In a first investigation of walking frame use in home environments**, each participant’s percentage incorrect use was calculated and graphically visualized for both pick-up walker and front-wheeled walker in the home environment (i.e. residential home, care home, sheltered housing). The associated device loading was computed for the front-wheeled walker (but not for the pickup walker, as the definition of incorrect use implies that the pickup walker is airborne during incorrect use). The videos taken in home environments were examined by the researcher to identify the context(s) in which the frame was used not in accordance with guidance.

**In a second investigation concerned with stability of the user-device system**, each participant’s average SM_min_ was determined for single and dual support during both correct and incorrect use. Where correct use was more often the case than incorrect use (or vice versus) in a given participant, a subset of SM_min_ values was randomly selected (within Matlab®) so that equal numbers of SM_min_ values for correct and incorrect use were used to calculate the participant’s average SM_min_. This was done for both, single and dual support. All SM_min_ data were normally distributed, and hence a general linear mixed effects model could be used for their analysis. In a first statistical analysis, the SM_min_ data obtained for single support during pick-up and front-wheeled walker use were analysed in SPSS; ‘Correctness’ (i.e. correct versus incorrect as per guidance) and ‘Environment’ (ADL flat, gait laboratory) were modelled as fixed effects. Initially the interaction term ‘Correctness x Environment’ was also modelled as a fixed effect but was found to be insignificant and was hence removed from the analysis. The individual was modelled as a random effect since participants physical abilities (and hence their stability margins) may be different due to, for example, age and/or co-morbidities. In a second statistical analysis, also using SPSS, the SM_min_ data obtained for dual support (front-wheeled walker use only) were also analysed with the same general linear mixed effects model.

### Qualitative work

Two focus groups [16] were conducted, one with walking aid users, the other with clinicians. The focus group members were all familiar with the use of commercial walking frames and use of our Smart Walker. The first group included five purposively recruited walking aid users (4 female & 1 male, age (mean ± SD) = 70.3 ± 4.8, body weight (mean ± SD): 79.1Kg ± 25.2Kg, Falls Efficacy Scale (mean ± SD): 43.3 ± 10.3), all of whom had participated in the experimental work of the study and therefore had met the associated inclusion and exclusion criteria. Each of them had had experience with between 2 and 4 different types of walking aids in their everyday lives. Participants either used multiple devices for different tasks, or had progressed from one device to another, either as a result of recovery from a fall, or because of further decline in their mobility. Walking aids had been obtained through a number of sources: from hospitals, nurses, community equipment stores, social services, but also from charity shops, bought online by a relative, passed on from a relative, ordered from Argos, and obtained through a Tombola. Since recruitment was limited by the co-morbidities of the research participants, i.e. cognitive ability to participate in a focus group and being mobile enough to travel to a central location, the group’s feedback was further substantiated by questionnaire feedback from nine additional walking aid users who were not able to attend the focus group (8 female & 1 male, age (mean ± SD) = 83.8 ± 4.1, body weight (mean ± SD): 82.4Kg ± 13.8Kg, Falls Efficacy Scale (mean ± SD): 43.7 ± 16.3, all users of front-wheeled walkers and 2 also using a pick-up walker). The second focus group included ten healthcare professionals with experience in prescription of walking frames (2 physiotherapists, 1 assistant practitioner working in nursing and residential homes, 1 occupational therapist, 2 discharge physiotherapists, 1 community physiotherapist, and 3 physiotherapists working in falls teams/services and supportive discharge). Their inclusion criteria was to be familiar with walking frames through regular exposure in their job as clinicians or other health care professional who supports users.

It was the role of an experienced focus group leader to moderate, facilitate and enable a lively and productive discussion, probe for details, and ensure input is received from all group members. A general opening question regarding users experience with walking frames was followed by a set of more specific trigger questions used to explore, for example, their experiences of using the smart walker (walking aid users only), its usability (both groups), and their willingness to engage with the technology (both groups). The healthcare professionals’ focus group was shown videos of the home assessments at the start of their focus group, to set the context as to how walking frames are used outside the clinic and what Smart Walker assessments entail. Both focus groups were audio recorded and subsequently transcribed verbatim; and the resulting data were processed using thematic analysis [17].

Further details on focus group members, questions asked, and detailed analyses, as well as the complementary questionnaire used, can be found in Additional file 1.

## Results

### Use of walking frames in home environments

#### Incorrect use of pickup walkers at home

Investigation of the percentage incorrect use of the two pick-up walker users assessed at home indicated that both users at times did not adhere to guidance: P01 did not adhere to guidance in 84% of single support periods, whilst P04 did not adhere to guidance in 16% of single support periods.

#### Incorrect use of front-wheeled walkers at home

**Fig. 3:**
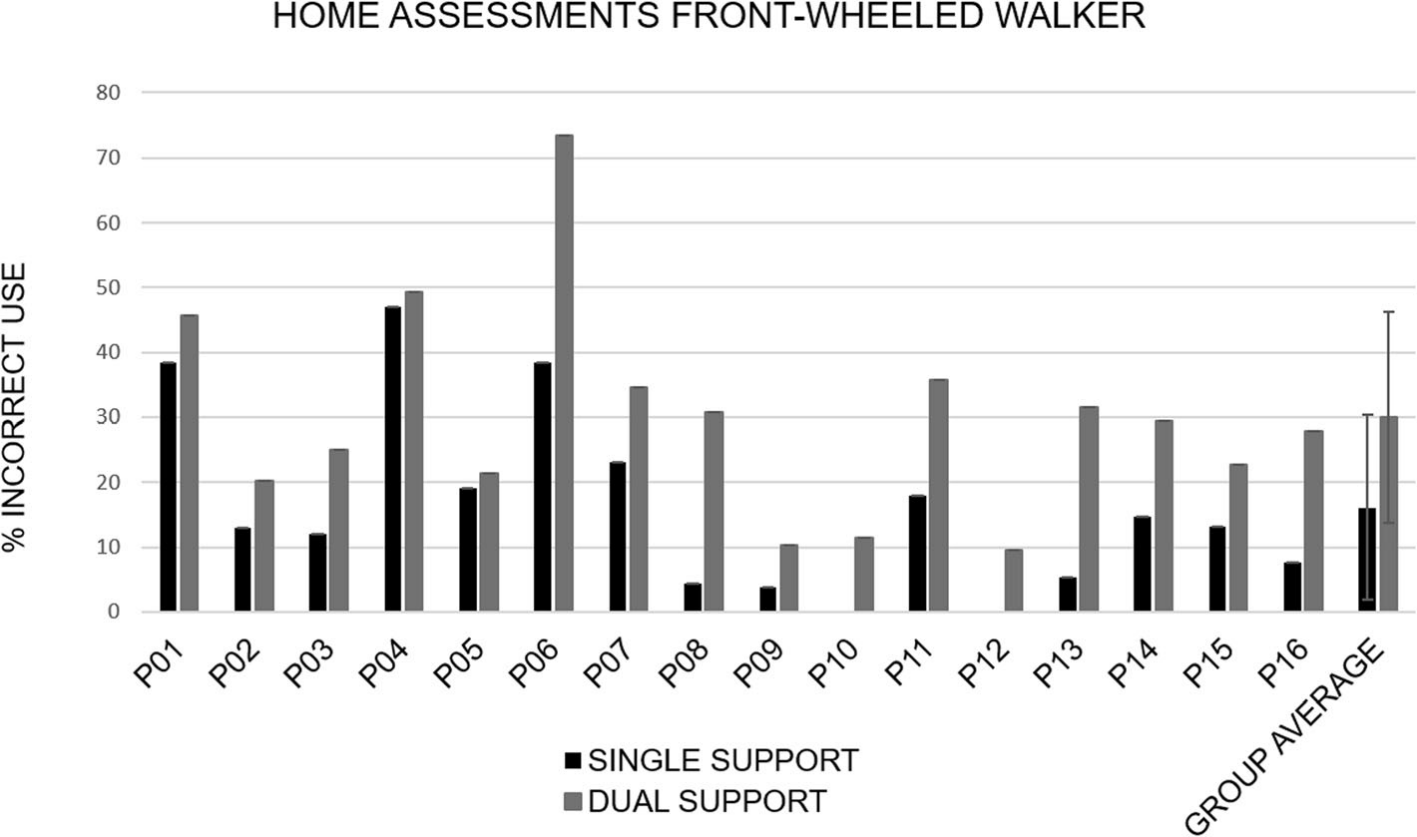
Percent incorrect use of the front-wheeled walker at home, defined as % single and dual support periods where one or both wheels of the front-wheeled walker were not grounded

**Fig. 4:**
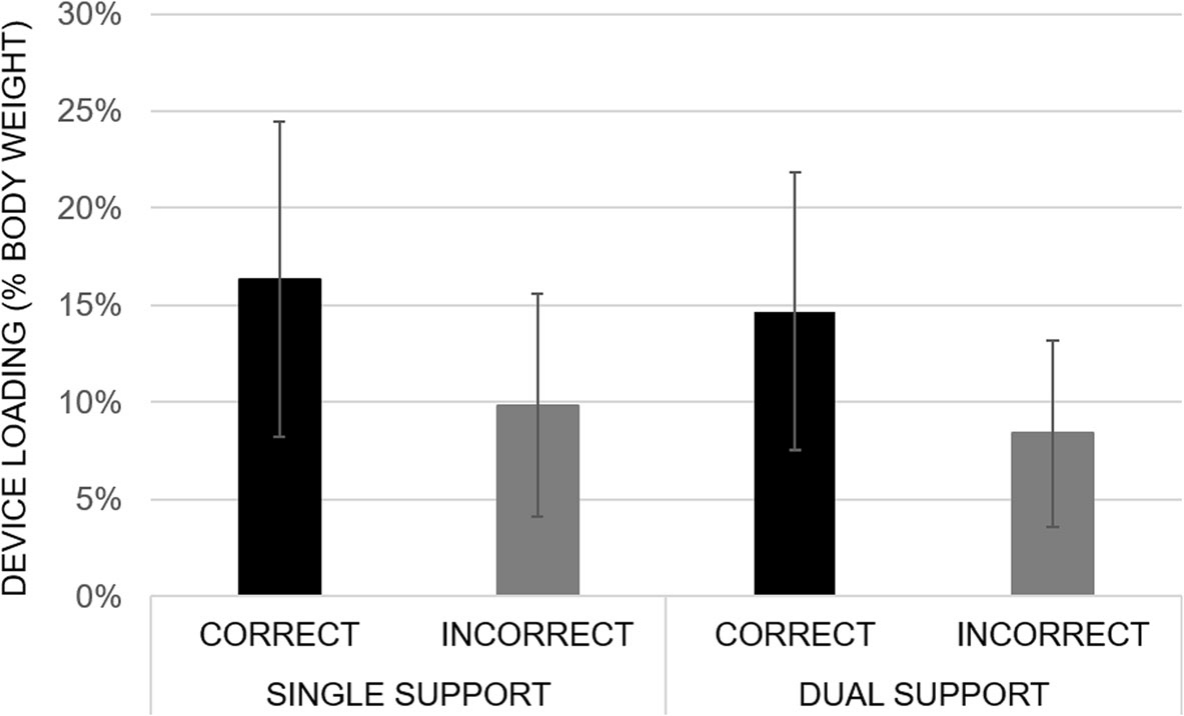
Average device loading for correct and incorrect use during single and dual support quoted as % body weight placed onto the front-wheeled walking frame, at home. Device loading is reduced for incorrect use, i.e. when one or both wheels of the frame are lifted off the ground

Figure 3 shows that the % incorrect use varied between participants, however, it is noteworthy that all participants exhibited incorrect use at some time. On average, the group had incorrect use during 16% of their single support periods, and 30% of their dual support periods. We note that the % of incorrect use is greater during dual as compared to single support for all 16 participants. The probability of this happening by chance is (1/2)^16 = 0.000015, i.e. 0.003% (probability multiplied by 100, for a two-sided test) and is significant. Subsequent secondary investigation of the average amount of body weight placed onto the front-wheeled walker revealed that device loading was greater for steps taken correctly, i.e. where both wheels remained in contact with the ground, and this was observed during both, single support and dual support (Fig. 4).

#### Video analysis of the environmental context

Home environments were generally tight for space, and routes between rooms often required moving over carpet edges (threshers) and turning corners. Rooms were often cluttered with furniture, and in the case of care homes, communal areas were cluttered with walking frames left by residents. Confined spaces together with clutter appeared attentionally demanding and to elicit manoeuvres which often deviated from guidelines on safe usage. Moreover, for the front-wheeled walker, which has both front wheels fixed (i.e. the wheels cannot swivel) it appeared impossible for users to turn whilst keeping the wheels on the ground: carpet friction together with device loading can prevent the frame from ‘skid steering’ on its wheels to face a new walking direction. Users were observed to either completely lift the frame off the ground and then step to turn whilst unsupported (in fact, whilst carrying the frame), or the frame was spun on a single pivot point (one of its legs), resulting in near-collisions between the person’s feet and the frame’s rotating legs.

### Stability of the user-device system

**Fig. 5:**
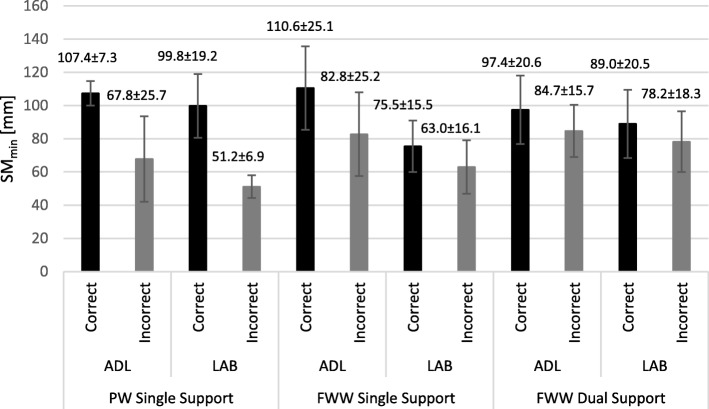
Group averages of SM_min_ values for walking in the ADL flat and lab environment. Shown are the average SM_min_ values during single support for the three pickup walker users (‘PW’), and the average SM_min_ values during single as well as dual support for the seven front-wheeled walker users (‘FWW’). A reduced stability margin can be observed for incorrect, as compared to correct, use across both types of walking frames and in both environments

Ten participants were assessed in both the ADL flat and the gait laboratory (3 pick-up walker users, 7 front-wheeled walker users). For the pick-up walker users, an average of 7 SM_min_ values per participant were observed and included in ADL flat data analyses, and an average of 21 SM_min_ values in gait lab data analyses. Participants’ average SM_min_ was lower for steps where the user did not adhere to guidance, i.e. was stepping whilst the frame was not properly on the ground with either 3 or 4 frame feet making contact, and this was consistently observed in both test environments, the ADL flat (37% reduction in SM_min_ for incorrect use) and the gait laboratory (49% reduction in SM_min_ for incorrect use), see also (Fig. 5). For single support of front-wheeled walker users, an average of 5 SM_min_ values per participant were included in ADL flat analyses, and an average of 8 SM_min_ values in gait lab analyses. For dual support of front-wheeled walker users, averages of 6 and 12 SM_min_ values per participant were included for ADL flat and lab analyses, respectively. Similar to the pick-up walker use, SM_min_ values for front-wheeled walker users were also reduced in both, single and dual support, for steps taken when the user was not adhering to guidance, i.e. was lifting one or both front wheels off the ground (Fig. 5). Specifically, lifting of the front wheels during single support was associated with a 25% decrease in SM_min_ in the ADL flat, and a 16% decrease in SM_min_ in the lab. Similarly, lifting of the front wheels during dual support was associated with a 13% decrease in SM_min_ in the ADL flat, and a 12% decrease in SM_min_ in the lab. Consistently, across both types of walking frames, across users, and across the two test environments, walking stability, as defined by the minimum stability margin, was lower for steps taken not in accordance to guidance (Fig. 5). Furthermore, we also observed reduced SM_min_ values for lab, as compared to ADL flat, assessments.

Initially, pick-up walker and front-wheeled walker data were combined for analysis of incorrect use versus correct use, and of environment (ADL flat versus gait lab), during single support. Table 2 shows statistical results of SM_min_ during single support, revealing that estimates of fixed effects were associated with *p*-values of *p* < 0.001 for ‘Correctness’ (i.e. correct/incorrect use) and *p* = 0.002 for ‘Environment’ (i.e. ADL flat/laboratory). Subsequently, the analysis was repeated for the front-wheeled walker data alone, and only slight changes in p-values were observed: *p* = 0.005 for ‘Correctness’ and *p* = 0.001 for ‘Environment’ (Table 2). Finally, the dual support data of the front-wheeled walker alone were also processed; respective p-values were *p* = 0.008 for ‘Correctness’ and *p* = 0.044 for ‘Environment’ (Table 2). Since incorrect use of a pick-up walker is defined for single support only (‘stepping whilst the walker is airborne’), analysis of dual support of the pick-up walker was not assessed.

**Table 2:** Overview of SM_min_ results. Shown is the % decrease in the group’s average SM_min_ from ‘correct to incorrect’ as well as from ‘ADL-flat to Gait Laboratory’, together with associated p-values from the General Linear Mixed Effects Model. FWW: front-wheeled walker, PW: Pickup Walker, SS: Single Support, DS: Dual Support

Change from A to B	SM_min_ Single Support FWW & PW Combined		SM_min_ Single Support FWW		SM_min_ Dual Support FWW	
	% Decrease	P	% Decrease	P	% Decrease	P
Correct to Incorrect	29.36%	< 0.001	21.85%	0.005	12.93%	0.008
ADL flat to Gait Lab	24.05%	0.002	28.59%	0.001	8.70%	0.044

### Focus group results

The following themes emerged from the walking aid user group: 1) enabling mobility, 2) design issues, 3) training/guidance, and 4) usability & acceptability of the Smart Walker system (supporting data in Additional file 1). Associated key outcomes were that walking aid use was clearly part of participants’ everyday life, suggesting frequent use and a positive impact on performance of activities of daily living and independence. However, concerns about safety came to light, highlighting the value of any research that aims to facilitate safe use of walking aids for more effective prevention of falls. Lack of guidance in the safe use of walking aids was viewed to be a problem, which the Smart Walker technology has the potential to address. The Smart Walker system and associated data were generally perceived acceptable and usable, and users showed willingness to engage with the technology’s output.

The following themes emerged from the healthcare professionals’ focus group: 1) prescription choice, 2) training equals practice, 3) problems, 4) extent to which smart walker data reflects clinical observation & judgement, 5) usability of the smart walker and willingness to engage with the technology. Associated key outcomes were that no gold-standard, universally-accepted approach exists for the prescription of walking frames and training of users. Healthcare professionals rely on their clinical judgement regarding the user’s safety and gait performance, and also take into consideration the person’s cognitive ability to use it. Notably, when asked how they train someone to use a frame they responded that it is practice and repetition. Regarding the home environment, it was considered to bring challenges, and there was agreement amongst healthcare professionals that home assessments are important, however, at present only intermediate care teams offer this service. Finally, a set of Smart Walker output visualizations in combination with video were found to agree with clinical observations & judgement of safe use. The utility of the Smart Walker technology was viewed to be particularly high in rehabilitation settings, but potential was also seen for using the technology to train healthcare professionals, and to inform design. Issues with usability were raised, including portability and ease-of-use being important, thereby highlighting the need for further changes in Smart Walker design to facilitate clinical adoption.

Outcomes are reported in more detail in Additional file 1.

## Discussion

Most studies concerned with walking-aid-assisted mobility assessed standard gait parameters, for example [18–21], but without reporting on gait stability. Only a few studies focused on stable use of walking aids, and they interpreted movements and loading of the walking aid alone to then infer on the stability of the user [13, 22, 23].

This study significantly contributes to the existing literature as it provides a novel perspective to a previously under-researched issue: how older people use their walking frame in relation to a rigorous mechanical measure of stability, which considers the user and their device as a single system. It is the largest biomechanics-based study of walking frame ambulation to date, and was conducted in both, laboratory and home environments.

In the home environment, use not in accordance with current guidance was found to be common for both pick-up walkers and front-wheeled walkers: all 16 participants assessed at home used their devices incorrectly at times. This suggests that users were either not provided with any clinical guidance on how to use their devices, or had forgotten/chose to ignore the guidance, or that other factors made using their walking frames according to guidance difficult or sub optimal. The analysis of video data lends support to the latter conclusion, showing that features of the environment as well as device design make it difficult, or in some cases impossible, to adhere to guidance. This questions the appropriateness of current clinical guidance for the home environment. Future work needs to review current guidance and walking frame design in relation to environmental constraints and everyday tasks other than straight line walking.

Regarding the front-wheeled walker specifically, video analysis showed that users lifted their frame when turning. Adoption of risky lifting strategies when turning were thought to occur because the frame’s front-wheels are fixed and do not swivel. This observation prompted the research team to review once more a number of clinical and manufacturer guidance documents and, alarmingly, guidance on how to turn safely are generally not discussed in these. Furthermore, door thresholds and carpet edges appeared to elicit lifting of the device. Other reasons not visible on this study’s videos may also contribute to the frame’s front wheels losing contact with the ground, for example when the person is losing balance in the posterior direction (i.e. “wobbling” backwards) and thereby tipping the frame backwards as a result. It may also be that being generally more upright is preferred, yet this increases the risk of the frame losing contact with the ground due to reduced device loading. We note that correct use of the front-wheeled walker, i.e. keeping its wheels on the ground, was associated with a greater load placed on the frame.

This study is the first to provide a set of objective, quantitative data on stability when using walking frames in both a home-setting (ADL flat) and a gait laboratory. We identified that stepping whilst a pick-up walker is not fully grounded, or whilst one or both wheels of a front-wheeled walker are airborne, reduces walking stability as quantified by the minimum stability margin SM_min_ of the combined user-device system. The decreased stability margin indicates that the system is closer to the point of “tipping over” when the device is not used in accordance with guidance. We propose that guidance should raise awareness of the challenges with turning, i.e. that the wheels of the front-wheeled frame do not swivel. As we have shown that lifting of the front-wheels reduces stability, in the short term, leaflets could suggest users should lift their frame as little as possible. Longer term, further work is needed to develop more detailed guidance and to improve frame design.

Considering that others previously reported gait to be less challenged in the lab environment as compared to the real world [13], it was surprising that SM_min_ values in this study were generally smaller in the lab environment as compared to the more realistic ADL flat. It may be that mapping out the ADL-pathway with tape on the laboratory floor, and thereby creating a somewhat abstract task within the lab, was cognitively challenging as participants had to monitor the tape-boundaries whilst walking, rather than walk naturally from one room to another (Fig. 1). Nevertheless, regardless of the test environment, incorrect use resulted in reduced SM_min_ values during single support (both devices) and dual support (front-wheeled walker). Future work needs to establish if poor stability relates directly to fall occurrence.

Qualitative work revealed the vital role that walking aids play in users’ daily life and highlighted that, despite the benefits, a lack of training and guidance in how to use them safely was of concern. The Smart Walker System was generally felt to be acceptable by users, and they saw potential for it to be used in training to improve how they used their walking aids. Comments from healthcare professionals suggested that they viewed practice and repetition to be training. This is interesting because by definition training is “the act of teaching a person a particular skill”, whilst practice is “repeated exercise in performance of an activity or skill” (Oxford Dictionary), yet healthcare professionals provided no detail as to the act of teaching walking aid use, i.e. for them training appeared to equal practice. Comments from healthcare professionals further highlighted that no gold-standard, universally-accepted approach exists for the prescription of walking frames and the training of users. They agreed that the Smart Walker System could be used to help address this. Moreover, the healthcare professionals agreed that the data generated by the Smart Walker were consistent with their clinical observations, and they saw potential for its use in rehabilitation settings as well as for training new healthcare professionals.

Although this is the largest biomechanics-based study of walking frame ambulation to date, generalizability of findings to the wider older population of users of walking aids remains limited by the sample size and the sample’s gender imbalance. It was generally challenging to recruit older walking aid users to the study because walking aid users are especially frail and suffer from cognitive decline. This also reduced participation in laboratory and ADL flat assessments for which participants had to come to the university. Moreover, the higher life expectancy of women likely contributed to gender-imbalanced sample. Another limitation of this study was that the majority of the data were obtained for walking with a front-wheeled walker. This is likely due to front-wheeled walkers being more commonly prescribed in the UK and their users being generally physically and cognitively more able, hence facilitating their participation in research. Finally, although the video analysis would have benefited from a formal coding scheme and a second rater, the initial observations reported in this paper suggest an urgent need for a more in-depth study of this issue.

## Conclusions

In conclusion, there was generally poor adherence to current guidance on how to use a walking frame safely in the home. Environmental factors as well as one design feature (i.e. front-wheels that are fixed and cannot swivel) are barriers to adherence to guidance. Stability analysis of the user-frame system in both ADL flat and gait laboratory revealed that the stability margin, i.e. how close the system is to the point of tipping over, was reduced during incorrect use. This finding, in combination with the high incidence of incorrect use observed at home, suggests that users are putting themselves at risk of falling when using walking frames in home environments. Future work is needed to investigate the relationships between incorrect use, stability margin of the user-device system, and falls incidence in users. Current guidance and device design should be reviewed and extended with the aim of improving stability during a range of everyday tasks including turning. Furthermore, guidance should consider environmental constraints to facilitate stable and therefore safe use of walking frames at home. The relevance of this work concerned with stable use of walking frames lies in the rising number of users, their increased falls-risk, and the costs of falls and complements other work on non-use of walking aids [24].

## Supplementary information

**Additional file 1.**

## Data Availability

The datasets used and/or analysed during the current study are available from the corresponding author on reasonable request.

## Abbreviations

ADL: Activities of Daily Living
BoS: Base of Support
CoP: Centre of Pressure
SD: Standard deviation
SMmin: Minimum Stability Margin
