# Supplementary material for: Are older people putting themselves at risk when using their walking frames?
Source: BMC Geriatr. 2020 Mar 4;20:90. doi: 10.1186/s12877-020-1450-2 (PMC7057469; doi:10.1186/s12877-020-1450-2)
Supplement: Supplementary file 1 — Additional file 1. [file 12877_2020_1450_MOESM1_ESM.docx]

**12. Supplement 1**

**Details regarding qualitative work.**

To provide another perspective to the biomechanical analyses, the research team also conducted qualitative work which consisted of focus groups and questionnaires for data collection.

Data was analysed using a framework approach (Braun and Clarke 2006). The researcher read and re-read the transcript of the focus group discussion. Following this, they identified codes and then found themes based on experiences, perceptions, and emotions in the data. Themes were then analysed, reviewed for their credibility, and finally analysed to draw inferences.

The aim of this qualitative work was to gain insights into the use of walking aids in general, and specifically, the use of the Smart Walker System. The first focus group included 5 purposively recruited walking aid users (*age(mean±SD)=70.3±4.8, body weight (mean±SD): 79.1Kg±25.2Kg, Falls Efficacy Scale (mean±SD): 43.3±10.3)*, and their feedback was further substantiated by questionnaire feedback of 9 additional walking aid users (*age>70, body weight (mean±SD): 82.4Kg±13.8Kg, Falls Efficacy Scale (mean±SD)*, all of whom had participated in the experimental work of the study. Inclusion criteria for participation were: 1) able to walk household distances with a walking frame, however, 2) not able to walk such distances repeatedly without a frame, 3) able to understand written and spoken English, able to follow a two-stage instruction. Exclusion criteria were: 1) currently in hospital, 2) visual disorders not correctable by glasses. . The second focus group included 10 healthcare professionals with experience in prescription of walking frames. Their inclusion criteria was to be familiar with walking frames through regular exposure in their job as clinicians or other health care professional who support users. Associated focus group guides detailing the running of the focus groups and the trigger questions asked are included in Appendix A & B at the end of this report, and the questionnaire is included in Appendix C.

- 1. **Experiences and views of walking aid users (Focus Group 1 and questionnaire analysis)**

Five (5) experienced users of walking frames participated in Focus Group 1. The number was limited by the co-morbidities of the research participants, i.e. cognitive ability to participate in a focus group and being mobile enough to travel to a central location. Each of them had experience with between 2 and 4 different types of walking aids. Participants either used multiple devices for different tasks, or had progressed from one device to another, either as a result of recovery from a fall, or because of further decline in their mobility. Walking aids had been obtained through a number of sources: from hospitals, nurses, community equipment stores, social services, but also from charity shops, bought online by a relative, passed on from a relative, ordered from Argos, and obtained through a Tombola. The focus group members were most keen on sharing their experience with their own walking aids and also commented on the potential usefulness of Smart Walkers. The opinions and perceptions of the participants were grouped into themes which are discussed below, and have been further substantiated by questionnaire data from 9 additional participants:

Enabling Mobility

For all participants, mobility was of key importance and walking aids were viewed by individual participants as enabling this:

*“We are not ones to sit in.” and “I couldn’t get out without it” (P4)*

*“I need it… I wouldn’t be able to go outside or do anything without it. I cannot manage going without it. I rely on it, especially outside.” (P8)*

*“I use it because I need support.” (P5)*

*“I would not be able to go out at all without it, and I am not a person to sit at home, so it has been a life saver.” (P6)*

Walking frames were discussed as helping with (fear of) falling: *“We are terrified of falling… it’s real fear.”* (P6) (Do walking frames help?) *“Yes, they help.”* (P6), and three users emphasized that what they specifically liked about their walking frame(s) is that they facilitate independence: *“Independence, you can get out and about.” (P6)*  *“Yes.”* (P5)*, “Yes, independence”* (P8).

- Overall, focus group members thought positively about their walking aids, considered them to be enabling and making them mobile.
- This was further reflected in the questionnaire data, where 7 out of 9 users reported to use their frame most of the time, another using it several times a day, and only one person reported to be rarely using it. Moreover, 5 reported to use theirs when going for a walk, and all 9 reported to use it when moving around the home and when going to the bathroom.

Design Issues

Participants mentioned only a few issues with design of common commercial walking frames, and these included difficulties to fit them into the boot of some cars, “take up a lot of room” (P4), being *big, getting in the way* (P5) and being *“bulky”* (P8), *“heavy”* (P4&P8), and with regard to pick-up walkers specifically, that the required movement pattern inhibits mobility *“You are not mobile, are you. You are static, then you pick it up”* (P5). Whilst the design feature of a shopping basked on the frame was perceived useful *“You can find you can get a weeks shopping in one of those”* (P7), there were also concerns about falls and safety.  *“If I put something in it [basket] you know if it goes on one side it goes unbalanced,…, you have to be careful.”* (P4) (Does this happens often?) *“Oh Yes! You have to be careful”*. (P4)

Training/guidance

Two participants stated clearly that they had not received any guidance or training:

*“No, I just went out and used it until I got used to it.”* (P6)

*“None really.”* (P7)

The remaining three participants discussed that they had some contact time with a healthcare professional or carer when they received their walking frame, however, two remained vague as to whether any guidance had been given:

*“I had two girls come to my house…and take me to XXX to see if I could manage it.”* (P4)

*“My social worker took me to [community equipment store] and they let me walk round the street.”* (P8)

Only one recalled the guidance received: *“They come by the side of you and say pick it up, put it down, and tell you what to do kind of thing.”* (P5) (for the pick-up walker).

When the group was asked specifically if they had received any training to get over obstacles and to negotiate steps, ramps, or curbs all answered: *“No.”.* (P4, P5, P6, P7, P8)

- Guidance given, if any, seems to be brief and basic, and may potentially have been forgotten. Everyday challenges encountered in the environment are not discussed. These findings are in agreement with information provided in Trust’s clinical leaflets, which focus mostly on performance of basic walking with no detailed discussion of how to safely perform turns, and negotiate steps/curbs, and ramps.
- That guidance is scarce was further substantiated by the questionnaire data: 5 of the 9 participants had received no guidance or training, and one participant was only told how to hold the frame, but without further instruction on how to actually use it.

**Usability and acceptability of the Smart Walker System**

When asked what they thought of the Smart Walker, participants were generally fine using it, with one specifically commenting that they found the device “comfortable” to use. However, three participants commented that they would not be able to use it outdoors. This would appear to be a limitation in the Smart Walker design, especially considering that all participants viewed going outdoors as important, however, the walking frames included in this study are models designed for indoor use only, In related work, a smart outdoor rollator has already been developed and tested, but was not part of this specific project.

When asked what they thought about use of Smart Walkers to monitor how well they walk with their walking frame, all said that they would be fine if the device was used in such way, with one commenting that that would be *“…absolutely brilliant if it improves them* (walking aids and/or their users)*”* and *“That would be better for a lot of people definitely, because at the moment they just give you the frame.”* (P6), and a third stating that *“They just leave you to your own devices, once they’ve got you situated you don’t see them again.”* (P5) All state explicitly that they would not mind if in future Smart Walker output was shared with or directly used by their clinician or carer (P4, P5, P6, P7, P8).

- These statements showed that the Smart Walker technology was generally viewed to be useful and that use of Smart Walkers in clinical practice was considered acceptable.
- Questionnaire data which was filled-in right after use of the Smart Walker supported this further: only two considered the Smart Walker worse than their own frame, one did not comment, and 6 of the 9 participants rated the Smart Walker to be similar or better than their own frame, and accordingly found the Smart Walker similar or better in terms of stability.

**Focus Group 1 Summary of Findings:**

- **Use of walking aids clearly was part of participants’ everyday life, suggesting frequent use for a positive impact on performance of activities of daily living and independence. However, concerns about design and safety remain, highlighting the value of any research that aims to facilitate safe use of walking aids for more effective prevention of falls.**
- **Lack of training/guidance appears to be a problem. Smart Walker technology has the potential to provide an evidence base for more complex walking tasks to enhance clinical guidance and training as to safe strategies with which one should perform everyday tasks at home.**
- **The Smart Walker system and associated data are generally perceived acceptable and usable by end users that are users of walking frames. Willingness to engage with the technology’s output was indicated.**
  1. **Experiences and views of healthcare professionals (Focus Group 2)**

The second focus group included 10 healthcare professionals: 2 physiotherapists, 1 assistant practitioner working in nursing and residential homes, 1 occupational therapist, 2 discharge physiotherapists, 1 community physiotherapist, and 3 physiotherapists working in falls teams/services and supportive discharge. All of them had extensive experience with walking aids and shared their views on prescription and guidance in general, and also regarding the potential use of Smart Walkers as part of clinical practice. Their comments were grouped into themes which are discussed below, together with supporting quotes:

Prescription choice

The group discussed how they decide what frame to prescribe for a given person, and it became clear that no gold standard, universally-accepted approach existed:

*“We mostly use the wheeled frames, don’t we, rather than the ones that you lift.”*

*“I suppose it’s what clinical reasons say are the safest.”*

Regarding the previous statement, safety of the user was a main concern:

*“We will prescribe what is safest for the patients, because that’s the main concern…”*

*“You get patients that ask to have 4 wheels on it…, but it’s not safe to have that as the walking frame can run away from you.”*

*“You would obviously assess them as you find them and deem whether they are safe or not.”*

Moreover, restoring as much of a normal gait pattern as possible was viewed desirable, and all agreed that cognitive ability needed to be considered. Interestingly, they also discussed that they keep an open mind if a frame is actually needed: *“Do they actually need a frame? So is that the right thing? Should we be looking at improving their balance and getting them off the frame if at all possible, rather than just giving the frame and thinking that’s it.”*

- No gold-standard, universally-accepted approach exists for the prescription of walking frames. Healthcare professionals rely on their clinical judgement regarding the user’s safety and gait performance, and they further consider the person’s cognitive ability to use it.

Training equals practice

The group was asked how they train someone to use a frame, and as for prescription, no standard protocol exists. Instead, simply practicing with the user was considered key:

*“Practice.”*

*“It’s just practice.”*

*“I’ve been doing a lot of practicing turning.”*

*“It’s practice and repetition.”*

*“… educating the patient as best as we can as to the best way of doing it, and that’s through repetition.”*

Regarding how much body weight a user should place onto their walking frame, comments were mixed: in some cases it does not matter, in other situations, for example when a patient is only partially weight-bearing after a fracture or if they are not picking up their feet well during walking, they would be encouraged to transfer more body weight onto the frame. However, too much weight on the frame was viewed to inhibit turning. In general, the participants encourage an upright posture, i.e. “standing tall”, proper weight transfer from the left to the right foot during walking, getting up from a chair by pushing off the chair (not pulling on the frame), and when sitting down, to move backwards with the frame until the rear of the legs can feel the chair. With the wheeled walker, a continuous walking pattern was encouraged: *“We’d encourage stepping and pushing rather than push, step, step, push, step, step.”*

- No gold-standard, universally-accepted approach exists for training users in the safe use of walking aids. However, all agreed that practice is paramount to learning to use a walking frame.

Problems

The focus group members demonstrated awareness of problems arising in the home environment:

*“There are environmental concerns as well, sometimes they haven’t even got the space to turn the frame around.”*

*“It can be a problem, environmental issues, space, clutter.”*

*“I mean, just a regular sized bedroom when you open the door sometimes you have a double bed there is not a lot of room. People have to abandon the frame, the frame goes so far then they use the table, the bed,…”*

*“In the hospital environment you’ve got nice wide corridors, nice flat surfaces, people will move out of your way…you are obviously not going home with the patient… all of a sudden the frame is too big, it’s too wide, can’t go through things.”*

*“Thick carpets…” “Dogs, cats…”*

Home visits were said to be important therefore, however, at present these happen only in intermediate care. Yet, not every person with a walking frame will be seen by an intermediate care team.

Other problems discussed related to design and manufacture such as slippery and/or uncomfortable handles leading to disuse of the frame, and wear and tear of the rubber feet (ferrules). Finally, and alarmingly, all members of the focus group agreed that tripping up over the frame/getting entangled in the frame can be an issue.

- Healthcare professionals demonstrated good awareness of the complexity of the home environment and were in favour of home assessments, which at the moment are only routinely carried out by intermediate care teams.
- Tripping up over the frame/getting entangled in the frame was raised as an issue with direct relevance for falls-risk.

Extent to which the Smart Walker data reflects clinical observation & judgement

The group was first shown a set of videos of participants as they were walking in their home. Subsequently, the same videos were viewed but this time synchronized with, and displayed next to corresponding Smart Walker output. Overall, the group engaged well with the Smart Walker visualizations, and they did not disagree with the Smart Walker output in relation to video:

*“I suppose this is what we have seen.”*

*“Suppose that confirms it statistically* [mathematically]*.”*

*“It is the evidence for what you are seeing.”*

Usability of the Smart Walker and willingness to engage with the technology

One focus group member commented that the Smart Walkers may not be useful during initial prescription of a walking frame:

*“I wouldn’t say for prescription though, I think I wouldn’t look at something like that and decide if someone needed a frame. I mean, the frames are so bog standard, if you had a whole range of frames that did different things then you would need the data. But you are prescribing the same bog standard frame, so your options are limited.”*

However, the group very much engaged in a discussion as to how Smart Walkers may be used for training and monitoring: unanimously the group acknowledged that currently no tests are done to see how someone is progressing, i.e. at the start and end of prescription and/or training. The stepping pattern as well as pressure measurements were perceived as useful to their practice in that regard. Comments supporting utility of Smart Walker technology in clinical practice included:

*“Falls patients sometime just say ‘oh I had 3 falls’ and you got no idea, but would love to know why…* [Smart Walker information] *would be useful if you saw that, you could say ‘well I can see why’.” “Yeah, you are tilting backwards.”*

*“I think so, yeah* [the stepping pattern would be useful]*. It would give us a bit more indication of what areas we can potentially work on.”*

*“And with amputees you could use this* [the stepping pattern] *as an outcome measure.”*

*“It would be useful for people with a discrepancy, maybe stroke patients who have a discrepancy between left and right, a marked one.”*

*“You could use it in training, you could actually show them this is how you are walking, this is what we need to work on.”*

*“Yeah, … improving awareness, I could see it in that sense, it would be quite useful.”*

*“And we’d always want to see a start, middle and end, we’d always want to see if there was a change. That’s why you’d use it in a rehab setting like stroke, you would want to see how people change.”*

*“It would be interesting to see if he* [person in video] *had a frame that we would suggest was his correct height, what the difference in the pressures and the stepping would be.”*

*“Yeah* [regarding the display of device loading in % body weight being useful]*, I would say potentially with falls patients.. there are a lot of instances where the weight is distributed towards the back of the frame, we could tell them to move forward.”*

*“I think that together with the wobble* [stability margin outcome measure] *that would be useful.”*

*“I think it* [device loading in % body weight support] *would be quite useful.”*

*“Yes (general agreement of the group).”*

*“If there was a standard of how much a person should be* [loading onto the frame]*.”*

And interestingly, comments were made in relation to use of Smart Walkers in training of healthcare professionals:

*“As a student as well, it would be great actually when you are studying your physiotherapy degree, it would be great to see some actual feedback. Something visible to say what is happening.”*

*“So connecting what they are seeing.”*

- Utility of the smart walker system was viewed to be particularly high in rehab settings, where supporting and hence monitoring of improvements over time is central to the work. Moreover, discussion of using the technology for training of healthcare professionals opened up a new route to impact that had not been previously considered by the research team.

Further potential for the use of Smart Walkers to inform the design of walking frames was revealed through the following comments:

*“I don’t know what research has been done to say ‘this is the optimum shape’. Everybody is a different shape and size, should they* [the walking frames] *be individual?”*

*“You can get slightly narrower ones… would a different base of support be better?”*

Final comments regarding usability and utility of the Smart Walker included:

*“Depends how difficult it is to use. Because we go in people’s homes, we’d have to bring the Zimmer […] in the car, or walking.”*

*“I think it would be impossible in the community setting. If you talk about intermediate care it might be useful.”*

*“In a hospital setting, say you had a stroke rehab unit or something like that, these things would be useful.”*

*“Yes!” (unanimous agreement that the Smart Walker would be more for a clinical setting)*

- The Smart Walker technology needs to be made more easily portable for community assessments and easy-of-use in general, for it to become an attractive tool for healthcare professionals in clinical care.

Finally, some commented that walking frames could be designed to prompt their users to “reach for the chair” , “stay close”, or “use me”.

**Focus Group 2 Summary of Findings:**

- **No gold-standard, universally-accepted approach exists for the prescription of walking frames and training of users. Healthcare professionals rely on their clinical judgement regarding the user’s safety and gait performance, and they also report taking into consideration the person’s cognitive ability to use it.**
- **The home environment was considered to bring challenges, and there was agreement that home assessments are important, however, at present only intermediate care teams offer this service.**
- **A set of Smart Walker output visualizations in combination with video were found to agree with clinical observations & judgement of safe use.**
- **Utility of the Smart Walker technology was viewed to be particularly high in rehab settings, but potential was also seen for using the technology to train healthcare professionals, and to inform design.**
- **Issues with usability were raised, including portability and ease-of-use being important, thereby highlighting the need for further changes in Smart Walker design to facilitate clinical adoption.**

**In conclusion,** Smart walking aids have the potential to meet end-users needs through 1) informing clinical guidance under consideration of user-device stability, 2) enabling evidence-based training & monitoring of walking aid users in relation to stability, device loading, and usage pattern, and 3) informing design of safer next-generation walking aids. However, further simplification of the system is required for it to become an attractive tool for home and clinical settings.

**References**

Braun V and Clarke V. Using thematic analysis in psychology. Qualitative Research in Psychology 2006; 3: 77-101.
